# Effect of Diet on Preference and Intake of Sucrose in Obese Prone and Resistant Rats

**DOI:** 10.1371/journal.pone.0111232

**Published:** 2014-10-20

**Authors:** Frank A. Duca, Timothy D. Swartz, Mihai Covasa

**Affiliations:** 1 UMR 1319 MICALIS, Institut National de la Recherche Agronomique, Centre de Recherche de Jouy-, Jouy-en-Josas, France; 2 AgroParisTech, Jouy-en-Josas, France; 3 Department of Basic Medical Sciences, College of Osteopathic Medicine, Western University of Health Sciences, Pomona, California, United States of America; 4 Department of Human Health and Development, University of Suceava, Suceava, Romania; University of Leicester, United Kingdom

## Abstract

Increased orosensory stimulation from palatable diets and decreased feedback from gut signals have been proposed as contributing factors to obesity development. Whether altered taste functions associated with obesity are common traits or acquired deficits to environmental factors, such as a high-energy (HE)-diet, however, is not clear. To address this, we examined preference and sensitivity of increasing concentrations of sucrose solutions in rats prone (OP) and resistant (OR) to obesity during chow and HE feeding and measured lingual gene expression of the sweet taste receptor T1R3. When chow-fed, OP rats exhibited reduced preference and acceptance of dilute sucrose solutions, sham-fed less sucrose compared to OR rats, and had reduced lingual T1R3 gene expression. HE-feeding abrogated differences in sucrose preference and intake and lingual T1R3 expression between phenotypes. Despite similar sucrose intakes however, OP rats consumed significantly more total calories during 48-h two-bottle testing compared to OR rats. The results demonstrate that OP rats have an innate deficit for sweet taste detection, as illustrated by a reduction in sensitivity to sweets and reduced T1R3 gene expression; however their hyperphagia and subsequent obesity during HE-feeding is most likely not due to altered consumption of sweets.

## Introduction

The over consumption of highly palatable, sugar-rich, and energy dense foods is a salient environmental contributor to the increasing worldwide obesity rates. Under such conditions, individuals susceptible to obesity are at higher risks for excess calorie intake, which contributes to obesity. Indeed, animals prone to obesity (OP) are hyperphagic and have increased energy efficiency when maintained on a high-energy (HE) diet leading to marked increases in body adiposity [1]. This animal model is characterized by a polygenic phenotype that closely resembles human obesity, and is useful in unraveling the interaction of obesogenic feeding and genetics on weight gain and obesity [2]. While altered peripheral metabolism, deficits in neural signaling, and leptin resistance are all contributors to chronic over-consumption and obesity in this model [3]–[5], impaired oral and post-oral feedback likely play an important role in the development of obesity [6], [7].

Consumption of palatable foods, specifically sweet stimuli, is regulated by oral and post-oral detection of nutritive and non-nutritive sweeteners (for review see [8]). During the presence of both oral and post-oral feedback, rodents exhibit an increased intake for nutritive and non-nutritive sweet solutions [8]. In the presence of only oral detection, animals consume sucrose in a concentration dependent manner, even when sated [9]. However, post-oral signals are also capable of influencing intake as gastrointestinal infusions of nutritive sweet solutions stimulate intake of a non-caloric flavored solution [10]. Oral and intestinal chemosensing of various nutrients is regulated, in part, by nutrient responsive receptors located on taste and gut epithelial cells, respectively [11]. For example, the heterodimeric G-protein coupled receptor T1R2/3, which is comprised of type 1 taste receptor subunits (T1Rs) coupled to α-gustducin, is responsible for detection of sweet stimuli, as knock-out of either receptor in mouse models severely diminishes lingual detection of sweet stimuli [12], [13]. The T1R3 subunit of the sweet taste receptor also plays an important role in nutrient-induced intestinal signaling as T1R3 KO mice have decreased circulating glucagon-like peptide-1 (GLP-1), decreased glucose tolerance, and abolished sugar-induced sodium glucose luminal transporter-1 up-regulation [14]–[16].

In general, obesity is associated with reduced oral and post-oral detection of nutrients [6], [17], [18]. However, it is less clear if increased caloric intake is a function of generalized heightened avidity for palatable tastants. Findings from human studies are conflicting, but recent data suggests that obese humans display enhanced ‘liking’ and lower intensity ratings for sweet solutions compared to non-obese individuals [19], [20]. In animal models, responsiveness to sucrose, but not fat, was an effective predictor of weight and adiposity gain [21], and accordingly, genetically obese rats exhibit decreased oral and post-oral sensitivity to sucrose that is associated with overconsumption of sucrose solutions [22]. Furthermore, while chow-fed OP rats exhibit increased taste reactivity to sucrose, OP rats do not display differences in long-term sucrose preference or in brief-access tests compared to obese-resistant (OR) rats [23]. Therefore, the oral and post-oral contribution to sweet intake in the OP animal is unclear, as does the role of the susceptibility to obesity, the obesogenic diet, and the interaction of these components. Thus, the aim of the present study was to examine differences in oral and post-oral sensitivity to sweets between OP and OR rats during chow- and HE-feeding. To do this, we first examined intake and preference for various concentrations of sucrose solutions by employing a 48-h two bottle access tests. Secondly, to determine changes in oral taste sensitivity between the strains, we sham fed OP and OR rats increasing concentrations of sucrose solutions. Lastly, to determine if the behavioral findings were associated with alterations in sweet detecting elements, we examined expression of lingual sweet taste receptor obligatory subunit, T1R3, in OP and OR rats during chow or HE feeding.

## Methods

### Subjects

A total of 66 male 8-wk-old Sprague-Dawley obesity-prone (OP) and obesity-resistant (OR) rats (n = 38 per each phenotype), weighing 275 g and 210 g respectively, were obtained from Charles River (Kingston, NY) and used throughout all experiments. Animals were housed in individual clear plexiglass cages with bedding, except where noted, in a temperature controlled vivarium with a 12∶12 h light/dark cycle (lights on at 0600 h). Throughout experiments, animals had *ad libitum* access to food and water, unless indicated otherwise. After one week acclimation, OP and OR animals were divided into chow-fed (3.3 kcal/g; in kcal%: 62.1 carbohydrates, 11.4 fat, and 26.5 protein; RM3A(P), SDS Diets, Witham, UK,) and HE-fed (4.41 kcal/g; in kcal%: 51.4 carbohydrates of which 50% of carbohydrates is from sucrose, 31.8 fat, 16.8 protein; D12266B, Research Diets, NJ) weight matched groups (n = 19 per group and phenotype). After 4-wks of feeding on their respective diets, each of the 4 groups (OP chow, OR chow, OP HE, and OR HE) were further divided into three testing groups: real feeding (n = 7), sham feeding (n = 8), and control (n = 4). All protocols were approved by the Animal Ethics committee for animal experimentation (COMETHEA) Jouy-en-Josas and AgroParisTech and carried out in accordance with the European Guidelines for the Care and Use of Laboratory Animals.

#### Intake and preference of sucrose solutions

OP and OR rats (n = 7 per phenotype and diet) naïve to sucrose were used for real feeding tests. Throughout experiments, rats had ad libitum access to food and water from two plastic bottles with metal spouts. On experimental days, one bottle was filled with varying concentrations of sucrose. After four days of acclimation rats were tested for acceptance (volume of intake) and preference (% of total liquid intake) of increasing concentrations (0.01, 0.03, 0.1, 0.3, 1.0M) of sucrose *vs*. water in 48-h two-bottle tests. Each concentration was presented at 1500 h for two consecutive days with a 24-h washout period between each concentration. Bottles were placed at the center of the cage, with each spout protruding into the cage at identical lengths, and a 3–4 cm separation between spouts, with the position of bottles switched after 24-h. Sucrose solutions were freshly made with deionized water and presented at room temperature. To account for spillage, two identical bottles filled with test solutions were placed on an empty cage, spillage calculated and subtracted from the total intake. Food intake, accounting for spillage was measured throughout the experiment. Forty-eight hour total fluid and food intake were averaged and presented as daily (24h) values. Preference was calculated by dividing the total amount of sucrose by the total amount of fluid (water+sucrose) consumed in 48 h. Following all experiments, animals were sacrificed and fat pads (epididymal, retroperitoneal, and visceral) were excised and weighed.

#### Sham Feeding

OP and OR rats naïve to sucrose were fit with chronic gastric cannulae as described previously [18]. After recovery and attaining pre-surgical weights, rats underwent three training trials of 60-min sham feeding sessions with 0.03M sucrose solution, in the morning (0900 h) following an overnight (1700–0900 h) water deprivation. During testing, rats were briefly deprived of water (0900–1600) but not food, and were tested for 1 h (1400–1500 h) sham intake of 0.03M sucrose solution until a stable baseline was achieved. Afterwards, rats were sham fed one of three sucrose solutions (0.03, 0.3, and 1.0M) in random order, every other day, with each concentration tested at least twice. Sucrose intake was individually recorded every 5 min for 60 min. During sham-feeding, all tubes were verified for proper drainage to minimize any sucrose entering the intestine during testing and prevent post-oral stimulatory learning cues associated with sucrose.

#### Oral glucose tolerance test (OGTT)

Rats were tested for glucose tolerance after the 3-wk access period to sucrose by administering 50% glucose solution (2 g glucose/kg bodyweight) via oral gavage. Tail blood was sampled at 0, 30, 60, 90, and 120 min post gavage, and blood glucose was analyzed with a glucometer (One-Touch).

#### Tissue Collection

Lingual epithelial cells were collected from a separate group of naive animals (OP chow, OP HE, OR chow, OR HE; n = 4 each). After a 5-h fast (0900–1400), rats were placed under deep isofluorane anesthesia, and lingual epithelial cells were collected using enzymatic dissociation as described elsewhere [24].

#### Quantitative Real-Time PCR (qRT-PCR)

RNA was extracted using an RNEasy Fibrous Tissue Mini-kit (Qiagen, France) according to manufacturer’s instructions. For cDNA synthesis, 2 µg of RNA was reverse transcribed in a reaction volume of 60 µl, using a high-capacity cDNA kit (Applied Biosystems, Courtaboeuf, France). Samples were run in triplicate in a reaction volume of 20 µl using a StepOnePlus (Applied Biosystems, Courtaboeuf, France) RT-PCR system. Transcription levels of T1R3 were quantified using an inventoried Taqman Gene Expression Assay and Gene Expression Master Mix (Applied Biosystems, Courtaboeuf, France). Relative mRNA expression was quantified using the 2^−ΔΔCT^ method with β-actin as internal control.

### Statistical Analysis

For real feeding experiments, body weight and adiposity comparisons between groups were performed using two-way repeated measures (diet, strain) analysis of variance (rmANOVA). Differences between groups for sucrose preference, acceptance, and total caloric intake were subjected to three-way (strain, concentration, diet) rmANOVA. Total caloric intake data was presented as daily average of calories from total 48 h solid food and sucrose solution intake. For OGTT, blood glucose levels were analyzed at every time point by two-way (diet, strain) ANOVA. For sham feeding, total 60 min intake of sucrose (in ml) was subjected to three-way (diet, strain, concentration) rmANOVA. Values from qPCR were analyzed using two-way (diet, strain) ANOVA. For all studies, analyses of variance were followed by post-hoc Bonferroni tests, where appropriate. The confidence limit for statistical significance was set at p<0.05.

## Results

### Body weight and adiposity

**Figure 1:**
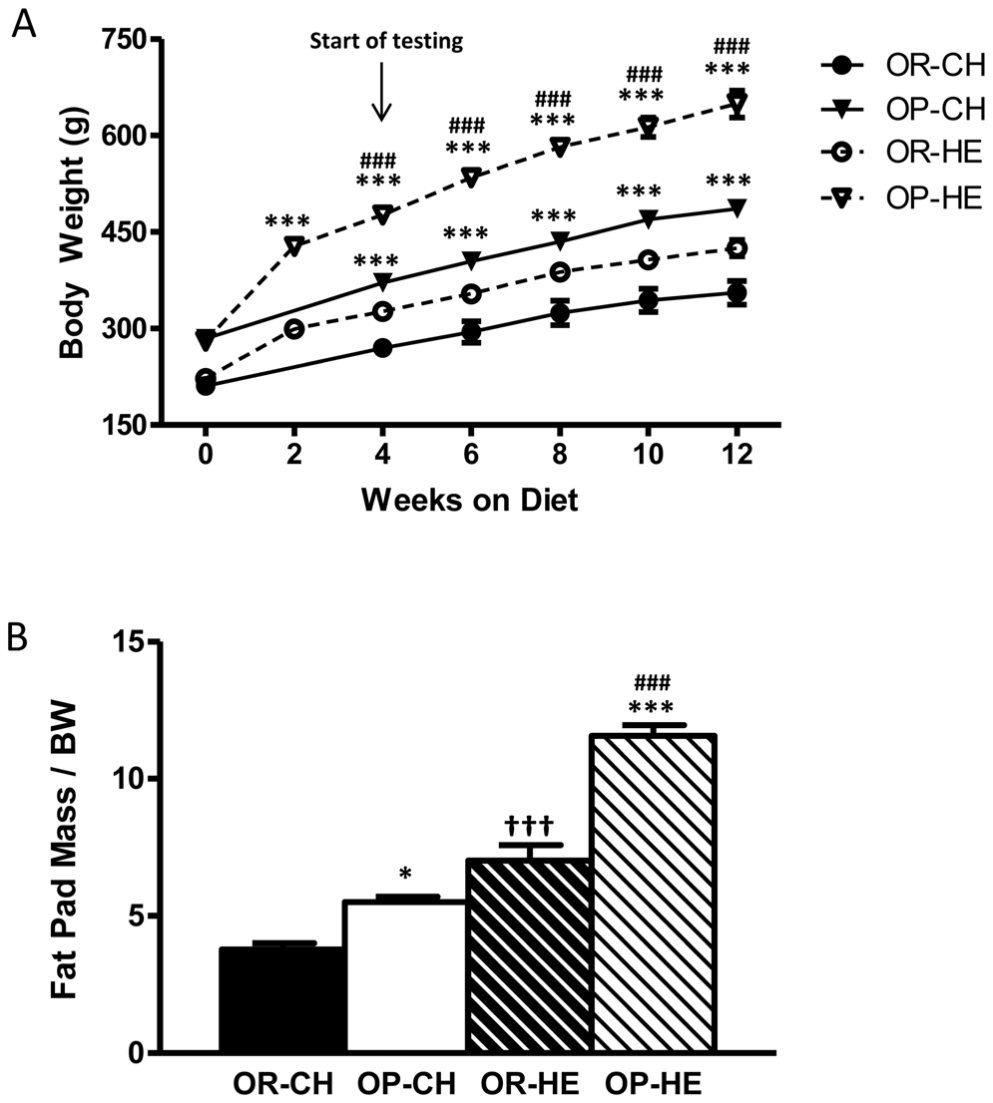
Body weights (A) and adiposity index (B) in chow- and HE-fed OP and OR rats. (A) OP rats weighed more than OR after 2-wks of HE-feeding and more than chow-fed OP animals after 4-wks. (B) Chow- and HE-fed OP rats had significantly heavier relative fat pad mass than OR counterparts, and HE-feeding resulted in increased adiposity of both OP and OR rats. Data are expressed as means ± SEM, *P<0.05, ***P<0.0001 OP relative to OR within diet condition; ^†††^P<0.0001 HE-OR relative to chow-OR; ^###^P<0.0001 HE-OP relative to chow-OP.

After 2-wk of HE-feeding, OP rats weighed significantly more than OR rats (P<0.0001; Fig. 1A) and after 4-wk of HE-feeding, HE-fed OP rats weighed more than chow-fed OP rats (P<0.0001). Total adiposity adjusted for body weight was slightly increased in OP compared to OR rats on chow-diet (P<0.05), while HE-feeding resulted in significant increases in adiposity in OP relative to OR controls (P<0.0001; Fig. 1B). Furthermore, HE-feeding increased adiposity of both OP and OR rats compared to their chow counterparts (P<0.0001 for both strains).

### Real feeding: sucrose preference and acceptance

**Figure 2:**
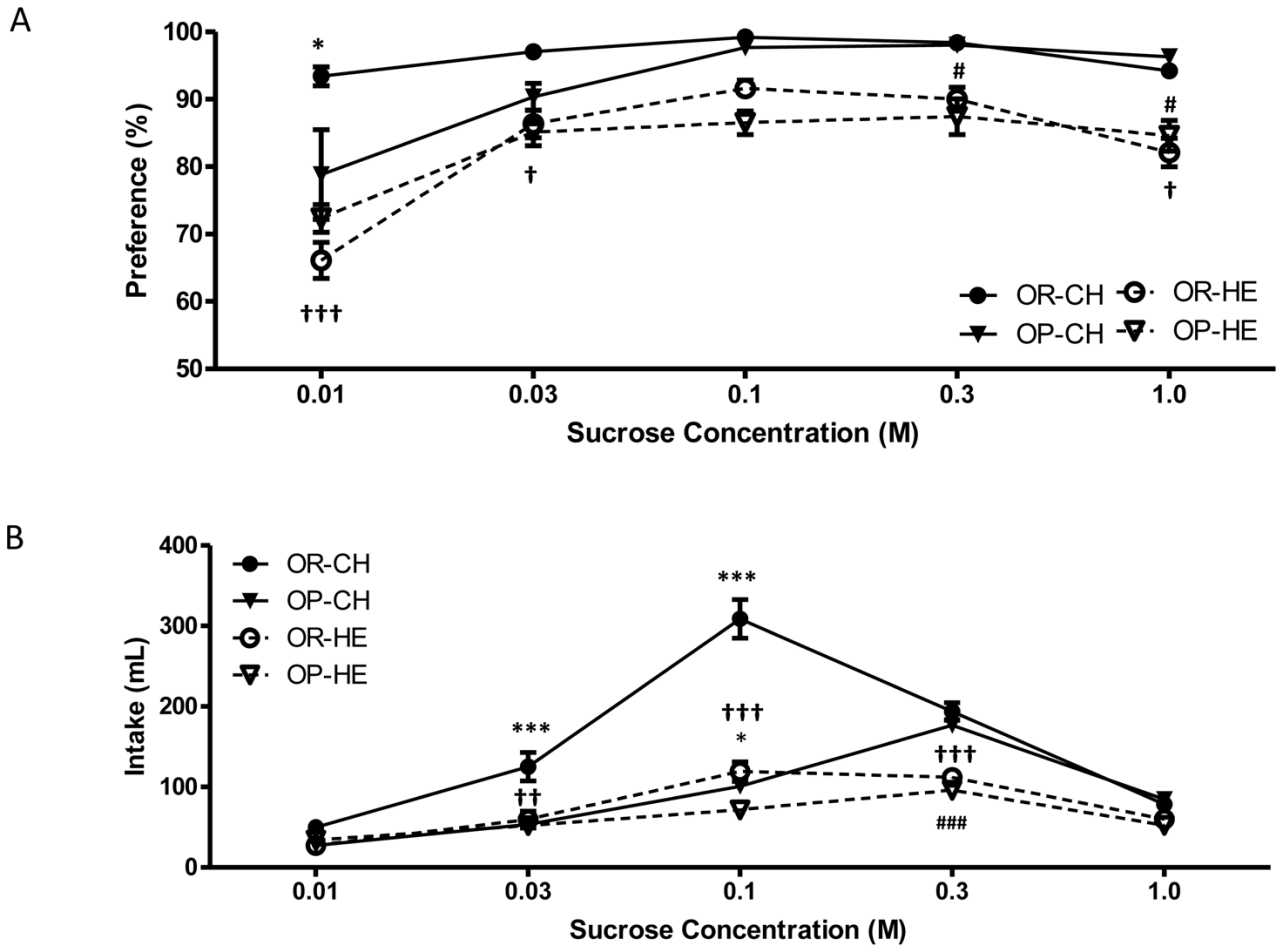
Preference (A) and intake (B) in OP and OR rats during 48-h two-bottle sucrose *vs*. water tests. (A) During chow-feeding, OP rats preferred less the lowest concentration (0.01 M) of sucrose than OR rats. Additionally, HE-feeding reduced preference of sucrose for both OR (0.01 M, 0.03 M, and 1.0 M) and OP (0.3 M and 1.0 M) rats. (B) Compared to OR rats, OP rats exhibited reduced intakes at 0.03 M and 0.1 M concentrations during chow-feeding, and at 0.1 M sucrose during HE-feeding. Furthermore, OP and OR rats consumed less sucrose when fed the HE diet compared to chow (OR at 0.03 M, 0.1 M, and 0.3 M; OP at 0.3 M). Data are expressed as means ± SEM. *P<0.05 OP relative to OR within diet condition; ^†^P<0.05, ^†††^P<0.0001 HE-OR relative to chow-OR; ^#^P<0.05, ^###^P<0.0001 HE-OP relative to chow-OP.

There were significant main effects of concentration [F(4, 81) = 60.08, P<0.0001], diet [F(1, 22) = 55.94, P<0.0001], and strain x concentration x diet interaction [F(4, 81) = 9.41, P<0.0001] on sucrose preference during real feeding. During chow-feeding, OP rats had diminished preference for 0.01 and 0.03M sucrose solutions with no differences between strains during HE-feeding (Fig. 2A). Compared to chow-feeding, HE-feeding resulted in an overall decreased preference for sucrose solutions in both strains. Specifically, OP rats preferred 0.3 M and 1.0 M sucrose solutions less during HE-feeding relative to chow-feeding (P = 0.046 and P = 0.012, respectively). As well, OR rats exhibited decreased preferences for 0.01 M, 0.03 M, and 1.0 M sucrose when HE-fed compared to chow-fed counterparts (P<0.0001, P = 0.043, and P = 0.013, respectively).

Strain [F(1, 23) = 36.9, P<0.0001], concentration [F(4, 88) = 227.40, P<0.0001], diet [F(1, 23) = 67.34, P<0.0001], and strain x concentration x diet interaction [F(4, 88) = 27.72, P<0.0001] all had a significant effect on sucrose acceptance during real feeding. Chow-fed OP rats consumed significantly less of 0.03 and 0.1 M sucrose solutions relative to OR animals (P<0.0001, for both) while HE-fed OP rats consumed less of a 0.1 M sucrose solution only (P<0.05; Fig. 2B), compared to HE-fed OR rats. Furthermore, compared to chow feeding, HE-feeding resulted in decreased intake of 0.3 M sucrose for OP rats (P<0.001), and a significant decreased intake of 0.03 M (P<0.01), 0.1 M (P<0.0001), and 0.3 M (P<0.0001) sucrose concentrations in OR rats.

### Calorie consumption during testing

**Figure 3:**
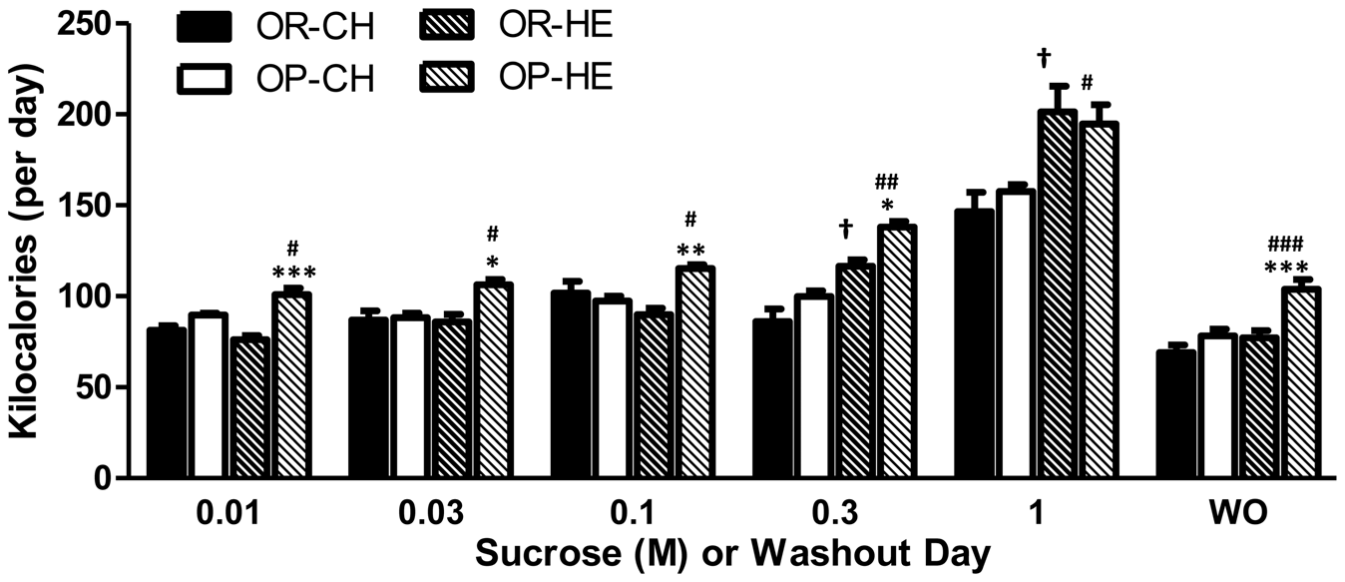
Total daily energy intake during 48-h two-bottle sucrose testing and washout days. During HE-feeding, OP rats consumed more total calories at all tests and washouts, except for 1.0 M sucrose, compared to OR rats. HE-feeding resulted in increased energy intake during tests of all sucrose concentrations for OP rats compared to chow counterparts. *P<0.05, **P<0.001, ***P<0.0001 OP relative to OR within diet condition; ^†^P<0.05 HE-OR relative to chow-OR; ^#^P<0.05, ^##^P<0.001, ^###^P<0.0001 HE-OP relative to chow-OP.

Although chow-fed OP rats consumed less sucrose solutions compared to OR rats, there were no difference in total daily calorie intake (calories from chow and sucrose) between OP and OR rats during sucrose testing or washout days. However, during HE-feeding, OP rats consumed significantly more calories than OR rats during all sucrose tests (P<0.05), except for the 1.0 M sucrose solution (Fig. 3). Furthermore, HE-fed OP rats consumed more calories compared to chow-fed OP rats at all concentrations (P<0.05).

### OGTT

**Figure 4:**
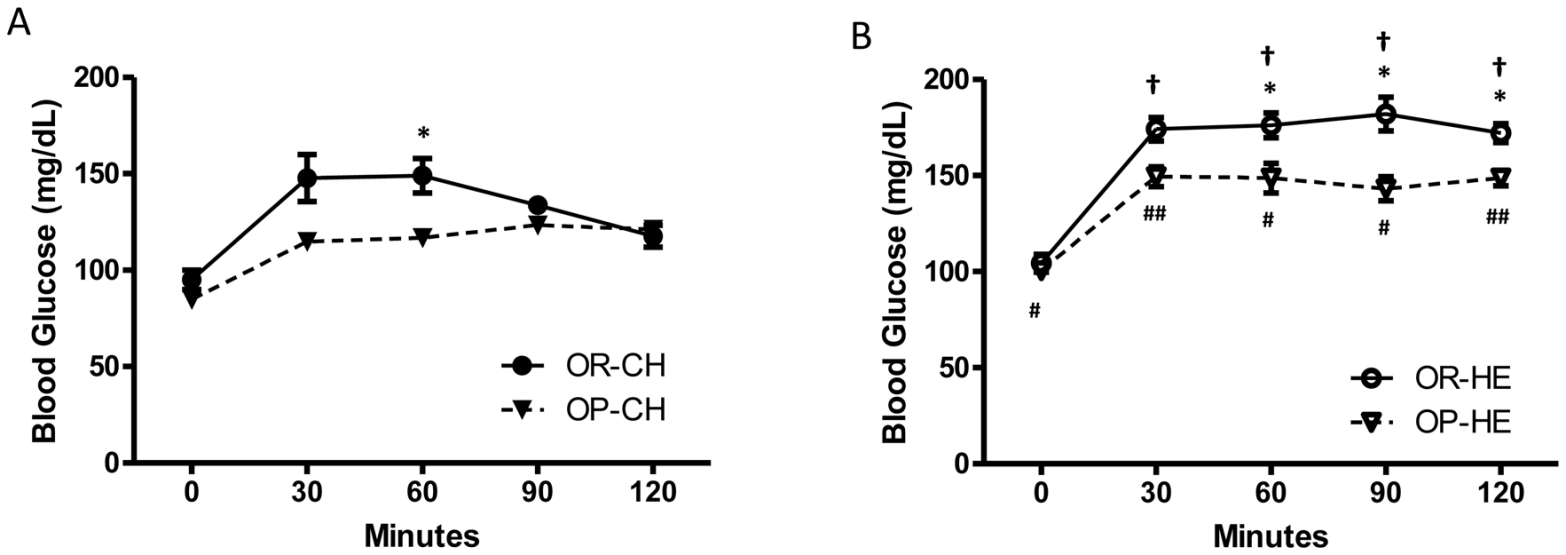
OGTT of OP and OR rats during chow- and HE-feeding. During chow-feeding, OP rats had reduced blood glucose levels at 60 min compared to OR rats, while HE-fed OP rats exhibited reduced glucose levels at 60, 90, and 120 min compared to HE-fed OR rats. *P<0.05 OP relative to OR within diet condition; ^†^P<0.05 HE-OR relative to chow-OR; ^#^P<0.05, ^##^P<0.001 HE-OP relative to chow-OP.

Fasting blood glucose levels were similar between strains during chow feeding (Fig. 4A). However, during HE-feeding, OP rats exhibited reduced blood glucose levels at 60, 90, and 120 minutes post glucose challenge (P<0.01, Fig. 4B). In both strains, HE-feeding led to elevated blood glucose levels at 30, 60, 90, and 120-min post glucose load compared to chow-feeding (P<0.05).

### Sham feeding

**Figure 5:**
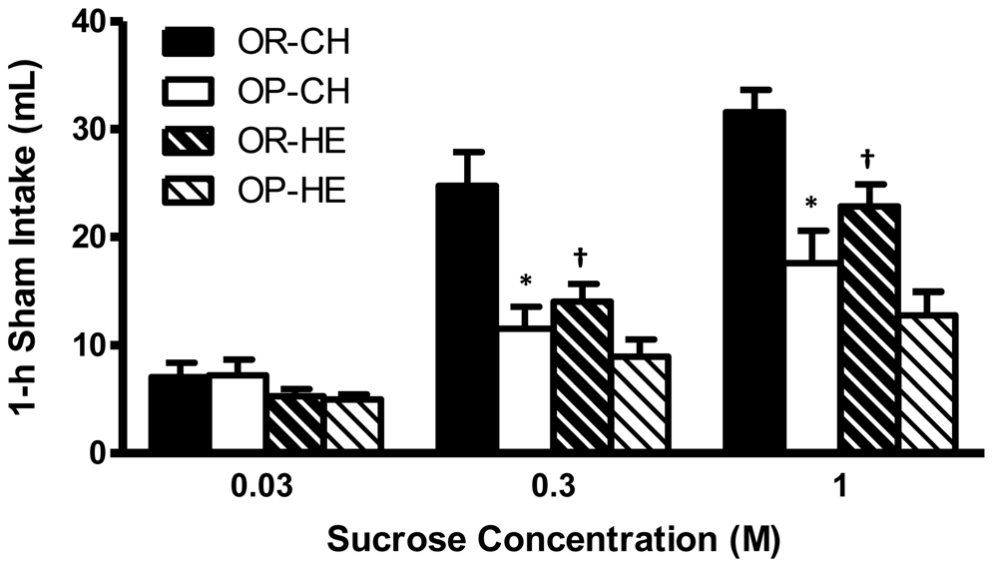
One-hour sham feeding intake of sucrose solutions for chow- and HE-fed OP and OR rats. Chow-fed OP rats had decreased intakes of 0.3 M and 1.0 M sucrose solutions compared to OR rats. HE-feeding resulted in decreased intakes of 0.3 M and 1.0 M sucrose solutions in OR rats. *P<0.05 OP relative to OR within diet condition; ^†^P<0.05 HE-OR relative to chow-OR.

Strain [F(1, 23) = 15.8, P<0.001], concentration [F(2, 120) = 60.13, P<0.0001], diet [F(1, 23) = 11.75, P<0.0022], and strain x concentration x diet interaction [F(7, 105) = 5.13, P<0.0001] significantly affected sham feeding intakes. Chow-fed OP rats consumed significantly less of 0.3 M (P<0.01) and 1.0 M (P<0.05) sucrose solutions compared to OR rats (Fig. 5), and HE-feeding abolished these effects (P>0.05; OP vs. OR, for each concentration). This was mainly driven by a significant reduction in 0.3 M sucrose and 1.0 M sucrose intake (P<0.05 for both) in OR rats during HE-feeding compared to chow-feeding.

### Lingual T1R3 mRNA Expression

**Figure 6:**
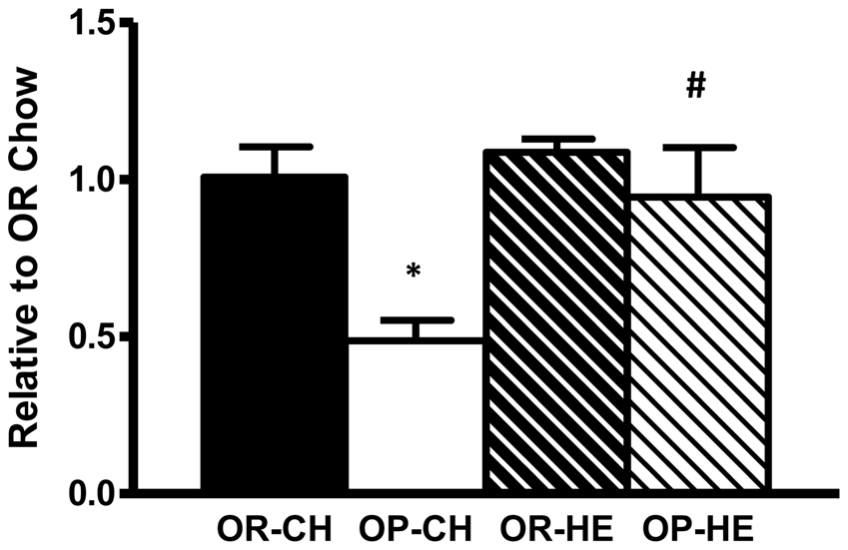
Gene expression of lingual T1R3 in OP and OR naïve animals OP rats had reduced T1R3 mRNA in the posterior lingual epithelium relative to OR rats during chow-feeding. *P<0.05 OP relative to OR within diet condition; ^#^P<0.05 HE-OP relative to chow-OP.

In chow-fed naïve animals, lingual epithelial T1R3 mRNA expression was significantly decreased in OP compared to OR rats (P<0.001), and this effect was abolished during HE-feeding (Fig. 6).

## Discussion

Our results demonstrate that chow-fed OP rats display reduced preference and intake of low concentrations of sucrose solutions during real feeding tests, decreased sham intake of sucrose, and diminished lingual T1R3 mRNA expression, all of which indicate decreased oral sensitivity to sucrose relative to OR rats. However, these effects are attenuated by HE -feeding, and despite similar levels of sucrose intake, OP rats still consume more total calories during 48-h access tests, likely indicating an overall deficit in inhibitory feedback during obesogenic feeding.

Overconsumption of sugary and energy dense foods is thought to contribute to the development of obesity in both humans [25] and rodents [1]. Whether increased caloric intake during obesity is a function of heightened avidity for sweets in most obese models has not been fully elucidated. Studies in humans are conflicting, with some demonstrating sweet taste sensitivity in obese subjects being similar to normal weight individuals [21], [26], while accumulating recent evidence shows that obese individuals rate sweets as less intense and maintain higher affinity for sweet stimuli relative to lean individuals [19], [20], [27], [28]. Similarly, both HE-fed and genetically obese rats exhibit an increased intake of high sucrose concentration solutions in the absence of diabetes [22], [29]. However, most of these studies are associative and do not provide causality for altered sucrose preference promoting obesity, with studies determining responses to sucrose in OP and OR rats remaining unclear. For example, some studies demonstrate a possible link between sucrose responsiveness and weight gain, while others show little or no differences in sweet preference between OP and OR rats [21], [26]. By employing a 48-h two-bottle testing paradigm during chow-feeding, we show that OP rats exhibit diminished preference and acceptance of dilute sucrose solutions compared to OR rats. This likely indicates inborn (genetic) deficits in oral or post-oral sensitivity to dilute sweet solutions in OP rats because these effects were observed prior to HE-feeding and the onset of obesity.

Short-term acceptance (volume of intake) and preference (intake relative to water) of sweet solutions is controlled predominantly by oral factors while long-term acceptance and preference for nutritive sweet stimuli develop from post-ingestive feedback, taste associations, and/or reinforcing oral cues [30], [31]. Thus, to elucidate the contribution of oral sweet sensing to our real feeding results, we demonstrated that chow-fed OP rats exhibit decreased sham intakes of 0.3 M and 1.0 M sucrose solutions. Sham feeding allows isolation of oral factors from post-oral feedback by limiting ingesta from entering the intestine and providing chemosensory feedback. Therefore, reduced sham intakes of sucrose strengthen our 48-h two bottle test results indicating that prior to HE diet exposure, OP rats have impaired sensitivity to sucrose. Our results of an overall decrease in sucrose preference and intake in OP rats are somewhat similar to the findings of Shin et al. who reported conflicting results in brief access (10 sec) licking and taste reactivity tests, both of which aim to assess the “liking” component of food reward [23]. As such, similar to our findings with sham-feeding, their chow-fed OP rats exhibited reduced liking for dilute, but not concentrated sweet solutions during brief access tests, however, OP rats displayed more hedonic reactions for highly sweet solutions in taste reactivity tests. However, both tests employed in that study generate brief and immediate responses to the test stimuli. This is in contrast with the two-bottle preference and sham feeding tests used in our studies that measure responses following longer, chronic exposure to the stimuli. Therefore, post-oral nutrient feedback (two-bottle test), habitual test stimuli exposure (sham feeding) or both may account for the lack of increased intake for higher concentrations of sucrose. Because taste is a weak predictor of day-long sweet solution intake, long-term acceptance and preference for nutritive sweet stimuli are primarily controlled by post-oral nutrient feedback that drives further consumption [31]. Intestinal T1R3, along with other taste signaling proteins (e.g. α-gustducin), are at least partially responsible for detection and ingestive responses to nutritive sweet stimuli [15], [16]. For example, T1R3 KO mice, which are unresponsive to sugars orally, consume less sucrose than control mice despite displaying normal preference for higher concentrations of sucrose solutions [12], [13], [32]. T1R3 is also expressed on enteroendocrine L-cells of mice and humans, and may contribute to glucose-induced GLP-1 release [15]. As such, we have recently found that chow-fed OP and OR rats have similar circulating and intestinal peptide levels of GLP-1 [33]. Furthermore, during the OGTT, which incorporates the intestinal sensing and hormonal signals in response to luminal glucose, we observed little difference between chow-fed OP and OR rats, indicating that post-oral responsiveness to glucose is likely normal in OP rats. Despite these indirect evidences, another hypothesis, which is not mutually exclusive from our current data, is that OP rats exhibit decreased post-oral learning of sucrose, in addition to decreased oral sensitivity identified via sham-feeding. As such, post-oral sucrose serves as a positive reinforcement leading to further sweet intake, which in this context, when decreased, could further decrease intake of sucrose in two-bottle tests (see [34] for review on post-oral flavor conditioning). Additionally, pathways involved in post-oral food reward and those involved in carbohydrate-induced satiation appear to be dissociated [34]. Therefore, while no differences in OGTT were observed between OP and OR rats, it does not rule out the possibility that pathways involved in intestinal conditioning of flavor preferences are altered. However, to date, no study has directly addressed the role of post-oral sucrose stimulation of intake and conditioning of flavor preference in OP and OR rats.

Lingual G-protein-coupled receptors T1R2 and T1R3 form a heterodimeric receptor complex to detect sweet tastants [12], [13]. Both subunits of the receptor are essential in sensing sweet substances as knockout of T1R2 or T1R3 results in a dramatic loss of sweet taste perception [13], and genetic variations in the genes encoding these receptor subunits are associated with differences in sensitivity to sweet taste in both rodents and humans [35], [36]. In agreement with our behavioral results, we found that naïve chow-fed OP rats display reduced T1R3 expression, similar to previous findings, showing decreased preference for saccharin in obese rats which was associated with reductions in lingual T1R3 gene expression [37]. Although we did not measure T1R2 gene expression, high-fat feeding and increases in body weight/adiposity result in changes in only T1R3 gene expression with associated differences in sweet perception, with no changes in T1R2 expression [37], [38], indicating that diet- and obesity-induced changes in sweet taste sensitivity are likely due to changes in the T1R3 subunit of the sweet taste receptor. However, whether obesity may have impacted sweet taste preference via T1R2 subunit cannot be concluded from this study. Taken together with our current results, these studies provide evidence that reduced sucrose consumption in OP rats may be a function of diminished gene expression of the T1R3 subunit. We did not examine a developmental time course of the reduction in T1R3 receptor expression, however, indirect evidence demonstrates that 4wk old OP rats exhibit reduced neural signaling pathways involved in taste [39], and 8wk old OP rats exhibit a reduction in “liking” of dilute sucrose solutions [23]. Together, these data may indicate that sweet taste deficits are present at an early stage in the OP rat model. While we did not measure neural activation following lingual stimulation with sucrose, these behavioral and molecular data suggest an inherited decreased oral sensitivity to sweet tastants before the onset of obesity.

In addition to possible differences in lingual sensory activation, reduced preference and acceptance of sucrose in chow-fed OP rats may be via differences in food reward. For example, OP rats fed a high-energy diet exhibit reduced central nucleus accumbens dopamine signaling, causing them to over consume palatable food to compensate for this deficit [40]. Additionally, OP rats display increased liking of high concentrations of sugars and fats [23], both of which are reversible with nucleus accumbens-targeted antagonism of mu-opioid receptors [41]. Thus, food hedonics can indeed drive palatable consumption, and studies suggest that food reward pathways, specifically central dopamine systems, may be blunted in the obese compared with normal weight subjects [42]. However, whether these differences are causal or secondary to obesity is debated [42], and while reversing obesity also reverses food reward functions, OP and OR rats show similar food reward differences before induction of obesity [23]. Despite this data, 4wk old OP rats exhibit central dopamine signaling deficits that could diminish the hedonic response in these animals [39]. Thus, more work is needed to determine whether food reward pathways play a role in our observed results [39].

Although our data demonstrate significant differences in sucrose preference and acceptance of OP rats compared to OR rats during chow-feeding, we found almost no differences in sucrose preference or intake between phenotypes during HE-feeding. This appears to be due to significantly diminished sucrose intakes predominantly in OR rats, as both sham and 48-h two bottle sucrose intakes were decreased for most concentrations tested in OR, but not OP rats. Therefore, it seems that OR rats are more responsive to the chronic exposure of the HE diet, that is also high in sugars, leading to reductions in sweet preference and intake [23], [37], [43]. The mechanisms responsible for the shift in oral sensitivity for sweets exclusively in OR rats following HE-feeding is unknown, but may involve a reduction in the intracellular signal transduction of the T1R2/T1R3 complex, changes in taste receptor cell number, and/or differences in upstream neural signaling pathways for sweet taste. For example, leptin is known to modulate sweet taste sensitivities as leptin receptors are localized on taste receptor cells expressing T1R2/3, and leptin inhibits mouse taste cell responses to sweet substances and reduces acceptance and preference of sweet solutions [37], [44], [45]. Therefore, it is possible that increased leptin levels in OR rats during HE-feeding (as adiposity was slightly higher compared to chow-fed OR rats) may be responsible for the reduction in sweet taste sensitivity. We can postulate that leptin resistance, typically exhibited by OP, and not OR rats, severely impairs and abrogates the effects of increased circulating leptin to diminish sweet taste sensitivity ultimately resulting in similar levels of sucrose intakes during chow- and HE–feeding in OP rats. On the contrary, OR rats, which remain leptin sensitive, may be more responsive to increases in circulating leptin, resulting in inhibition of taste cell signaling and subsequent reduction in sweet taste sensitivity, resulting in an overall decrease in preference and intake to properly maintain body weight.

During HE -feeding, despite maintaining similar sucrose intake compared to OR animals, OP rats consumed more total calories in real feeding tests. This was true for every concentration, except for the 1.0M, which is likely due to a ceiling effect. Interestingly, we observed that OR, but not OP, rats reduced intake of sucrose in the real feeding tests during HE-feeding compared to chow-feeding, resulting in a similar total caloric intake for most of the concentrations tested. This indicates that in the face of obesogenic feeding, OR rats are more effective in sensing ingested nutrients and adjusting subsequent intake to maintain energy homeostasis, while OP rats fail to do so, resulting in increased total caloric intake. Although OP and OR rats displayed no differences in liquid sucrose intake during HE-diet feeding, 51% of the energy from this diet is derived from carbohydrates, with half of this energy from sucrose. Therefore OP rats still consumed more total sucrose (liquid and diet combined) than OR rats during 48-h two bottle testing, perhaps indicating a reduction in the ability to detect luminal sugars. However, during the OGTT, HE-fed OP rats had a better response to the glucose load than OR rats, indicating that OP rats do not exhibit deficits in intestinal sweet sensing and response. Besides high sugar content, the HE-diet also has increased fat content, and it is likely that reduced intestinal sensitivity to fat is responsible for their overall increased intake. Indeed, high fat feeding is known to reduce intestinal sensitivity to fat loads in both rodents and humans [46]. This is associated with reduced protein expression of gut peptides and epithelial fatty acid sensing elements [17]. Furthermore, OP rats exhibit reductions in both CCK and GLP-1 levels and sensitivity during HE-feeding, both of which are secreted in the presence of luminal fat [33], [47]. Together, these findings, with our current data, implicate deficits in lipid sensing, rather than sugar sensing as the most likely cause of hyperphagia and subsequent obesity in OP rats. Indeed, these strains were created based on their feeding and weight gain response to a HF diet. Thus, it is plausible that increased caloric intake of OP rats during real feeding tests stems from their inability to sense lipids, likely from a reduction in gut peptide secretion and signaling.

In summary, these results demonstrate that prior to obesity, and during chow feeding, OP rats have decreased oral sensitivity to sweets, evidenced by diminished real and sham feeding of sucrose accompanied by decreased lingual T1R3 mRNA expression. However, when fed a HE-diet, these effects are largely abolished, with both OP and OR rats consuming similar volumes of sucrose. Thus, it is improbable that hyperphagia of OP rats is due to alterations in sweet taste. Rather, reductions in post-oral nutrient-sensing mechanisms for other nutrients, such as fats, likely play an important role in the increased caloric consumption of OP rats during HE-feeding.
